# iReach: new multisensory technology for early intervention in infants with visual impairments

**DOI:** 10.3389/fpsyg.2025.1607528

**Published:** 2025-05-20

**Authors:** Monica Gori, Stefania Petri, Martina Riberto, Walter Setti

**Affiliations:** Italian Institute of Technology, U-VIP Unit for Visually Impaired People, Genoa, Italy

**Keywords:** infants, development, multisensory, technology, visual impairment, intervention

## Abstract

Visual impairments have profoundly adverse consequences for infants. They affect infants’ spatial and motor skills, playing, socializing and psychological well-being. Early therapeutic interventions to foster these abilities are needed to improve their quality of life. Effective rehabilitation technologies depend on a better understanding of the neuroscientific bases of multisensory and body processing. However, these abilities can only be assessed qualitatively based on observational approaches. Technological solutions are still unavailable due to the complexity of conveying a signal to visually impaired infants that they will assuredly understand. With *iReach*, we aim to address this problem. Specifically, we will design and develop a multisensory system that will provide non-invasive recording and training of the sensory-motor skills. It will be compatible with simultaneous behavioral and brain activity response measures. For the first time, with *iReach* we will train and directly quantify visually impaired infants’ sensory-motor abilities, offering a cost-efficient system for early intervention.

## The problem

Vision is crucial for developing the skills to interact with objects and others. Impaired visual perception in infants with congenital blindness affects the development of reaching, locomotion and spatial skills, along with the way infants play and socialize with other individuals (Gori et al., 2008, 2010; Röder et al., 2004; Guralnick et al., 1996a; Guralnick et al., 1996b; McConnell and Odom, 1999; Rettig, 1994; Sacks et al., 1992). Overall, early visual deficit profoundly impacts infants’ physical functioning psychological well-being, and health service needs (Horowitz, 2004). Crucially, the visual system underlies the development of a multisensory spatial representation of the world, linking sensory inputs, motion, and body perception. This evidence is also supported by research about the role of visual input on multisensory space development with new methods of investigation [e.g., high-density electroencephalography (EEG)] (Bertonati et al., 2023; Campus et al., 2021). Sensory-motor interactions occur during the first years of life when cortical plasticity is maximal. For example, a multisensory representation of space emerges during the earliest years of development from the association between sensory-motor and body representations mediated by vision. Thus, early therapeutic interventions specifically aimed at fostering the development of sensory-motor abilities are needed to improve the quality of life of visually impaired infants. More studies are needed for identifying key developmental windows during which targeted support is most effective and for designing tailored interventions to enhance sensory-motor process skills in blind individuals from the first stages of life. Despite the well-known importance of early rehabilitative interventions, no studies evaluated their effects. This is mainly due to a lack of rehabilitative devices that are intuitive, simple and easily usable from the early stages of life (Gori et al., 2016).

**FIGURE 1:**
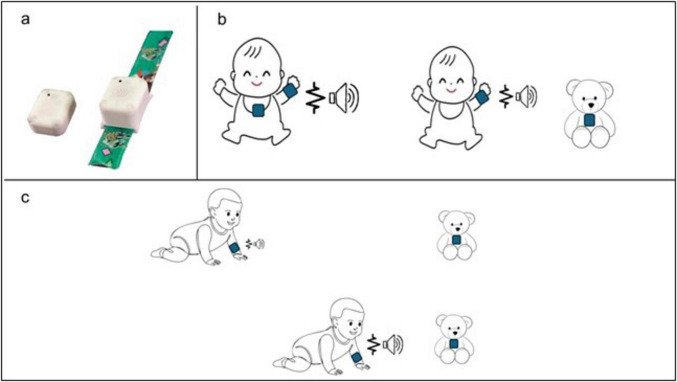
First prototype of the *iReach* system and the concept of functioning. **(a)** Image of the iReach units: the *Tag* on the left and the *Anchor* on the right; **(b)** different Tag positions: the Tag can be placed either on the infant’s body midline (left), or on external object (right); **(c)** example of the use of *iReach* in ecological contexts: the audio and tactile stimulation vary in intensity and in frequency with the distance between the Anchor (on the infant’s arm) and the Tag on the external object. For instance, as the infant crawls toward a toy with the Tag, he experiences gradually intensifying vibrations on the wrist and increasingly louder sounds. This dynamic multisensory feedback is designed to guide the infant’s exploratory behavior toward objects location, reinforcing spatial localization skills through active movement. The sound emitter and waveform icons represent the auditory and tactile stimuli, respectively. An increase in icons size indicates a corresponding increase in feedback intensity and frequency.

iReach mainly aims to develop a solution to restore the sensory-motor-body link and to improve motor and spatial skills when the visual modality is unavailable in infancy (framework illustration in Figure 1. Developing a technology designed for this purpose would fulfill a critical and still unmet necessity for basic and clinical research. However, several technological and methodological constraints have posed significant challenges to the development of multisensory devices for the spatial rehabilitation of infants with visual impairments. These obstacles include, but are not limited to, (i) the need to create a signal understandable to an infant (i.e., intuitive, without training), (ii) the need to produce a signal that does not require high attentional resources, still missing in infants, (iii) the need to understand the physiological mechanisms to be recovered, and (iv) the need to quantify the status and possible improvements of the infant after a short training session. For all these reasons, researchers have been unable to develop and validate a system to improve the sensory-motor and spatial skills of infants with visual impairment.

## The solution

We will design, develop, validate, and propose for commercialization the *iReach* system as a wearable device for easy, intuitive, and accurate stimulation of space and body representations for visually impaired and blind infants. This device is based on multi-sensory-motor signals provided in individual and social contexts. We have already developed the prototype of the system (Figure 1). *iReach* will deliver audio and tactile stimulation that will provide absolute information about the overall position of the arm and relative information regarding its position in relation to spatial reference. Specifically, it will consist of two units: a bracelet (Anchor), which contains a speaker and a vibro-motor and a reference unit (Tag) connected to the Anchor via wireless link (Figure 1a). The audio emitted by the Anchor will vary in intensity, while the tactile feedback will vary in frequency, both as a function of the spatial distance between the Anchor and the Tag, which can be positioned in different locations, both on the infant’s body or on external objects (Figures 1b, c). The audio-tactile stimulation parameters can be optimized to ensure the intuitiveness and effectiveness for spatial and sensorimotor training in visually impaired infants (for example, increasing their intensity). The association between the audio and tactile absolute and relative spatial representations will restore a unified sensory-motor-body representation to support spatial processing in blind infants. *iReach* will permit to recalibrate the bodily and spatial representations of visually impaired infants. It will also allow us to measure the infant’s responses and reaction times to stimulations in combination with EEG to determine the critical periods for early interventions. Specifically, we will plan an experimental procedure wherein infants will be involved in multisensory spatial perception and reach-to-grasp tasks, with and without the device, that will combine standardized clinical tests of motor abilities and EEG measurements. We will include only infants with visual impairments excluding those with additional motor or cognitive impairments.

To address infants’ ability to perceive and interpret multisensory stimulation, we will plan a dedicated usability testing phase before training. The usability tests will involve both blindfolded sighted infants and visually impaired peers: we will ask them whether they perceive changes in three types of stimulation: (1) Tactile and (2) auditory stimulation only in which they will vary in intensity, and (3) combined auditory and tactile stimulation in which simultaneous presentation of both modalities will change both in intensity and frequency. During this procedure, the Tag will be positioned on external object to evaluate spatial responses under different reference conditions. We expect the sensory feedback to be comprehensible to infants; however, if an infant does not understand the stimulation during the usability tests, we will postpone their inclusion until they acquire the necessary competencies. We will report the number of children who successfully pass the usability phase as well as the number of those who did not.

Practical considerations related to hardware design are also being carefully addressed. Currently, the wearable components of the iReach system are undergoing rigorous safety testing to ensure they meet the necessary standards for infant use. All safety protocols for wearable device will be fully satisfied by the time the system is implemented in the study, ensuring its suitability for both research purpose and future clinical application.

## The origin

The idea stems naturally from preliminary data of blind infants in restrained conditions performed within the ERC Starting Grant MySpace (agreement n° 948349). The goal of MySpace is to understand the mechanisms of audio-tactile multisensory spatial processing in blind infants and children. Through MySpace, we demonstrate the differences between blind and sighted infants in audio-tactile representations (Gori et al., 2021). Behavioral data have been collected with a device that conveys non-invasive audio and tactile absolute stimulations to visually impaired infants’ hands (Gori et al., 2019). Our findings show that, under incongruent audio-tactile stimulation conditions (i.e., tactile stimulation on one hand and auditory stimulation on the other one), blind infants exhibit a lower percentage of orienting response toward auditory stimulation compared to their sighted peers, demonstrating a stronger reliance on tactile than auditory cues when the two are presented in conflict. However, although blind infants show less overall gain from multisensory integration than to sighted peers, they reveal some forms of multisensory integration. This is evident in the differences in reaction times (RTs) between blind infants and sighted peers, where blind infants demonstrated lower RTs in the audio-tactile condition than in the auditory and tactile unisensory condition. This might suggest that they tend to integrate spatially redundant tactile and auditory cues to speed up their performance, rather than processing them independently. This supports the potential effectiveness of multisensory stimulations in the absence of vision (Gori et al., 2021).

### Demonstration of breakthrough innovation potential

The *iReach system* will provide tangible benefits at three levels:

(1) Impact on the economy. *iReach* will result in producing the first-ever science-based rehabilitation technology for infants with visual impairment. This is a particularly urgent need because there are currently no multisensory technological rehabilitative solutions for early intervention in blind children (Cuturi et al., 2016; Gori et al., 2016). Rehabilitation and support for visually impaired individuals costs 56.52 billion euros in total for the visually impaired population (Chakravarthy et al., 2017). The annual global costs of productivity losses that are associated with the treatment of uncorrected myopia and presbyopia alone were estimated to be US $244 billion and US $25.4 billion, respectively. Moreover, it has been estimated that the average annual cost of potential productivity losses due to moderate to severe visually impaired and blind employees is $410.7 billion (0.3% of US Gross Domestic Product).^1^ These costs can be significantly reduced by providing visually impaired infants with early intervention and scientific-based training to improve their mobility, social skills, and independence, which could be done using *iReach*. At present, there is only an audio solution for children from 3 years of age children called ABBI (Cappagli et al., 2019; Gori et al., 2016), which we have developed within the FP7 program grant agreement. However, ABBI provides only auditory absolute information, and therefore it lacks the possibility of improving tactile perception and multisensory audio-tactile integration. In turn, *iReach* will be an unconstrained, user-centered rehabilitative technology based on multisensory feedback with incredible potential benefits in both research environments and clinical practice. These reasons underline a clear market need for intuitive, non-invasive, ecological, and cost-effective systems for early-intervention and for monitoring visually impaired infants.
(2) Impact on society. *iReach* will become an efficient and inexpensive tool to explore and rehabilitate the spatial and body skills in infants with visual impairments. Children with early-onset severe visual impairments experience delayed motor, language, emotional, social, and cognitive development experiencing lower levels of educational achievement (Ryles, 1996; Tobin et al., 1997). In addition, visual impairments severely affect the quality of life among adult populations. Indeed, visually impaired adults often have lower rates of workforce participation and productivity and higher rates of depression and anxiety (Evans et al., 2007), increasing social isolation. Despite the significant advances in understanding the behavioral and cortical mechanisms of multisensory processing in blindness, remarkably in the last decade little has changed in the evaluation and treatment of visually impaired infants. The technological solutions developed so far are lowly accepted by visually impaired adults mainly because of their complexity. Moreover, they are not suitable for visually impaired children and infants. Therefore, about 2.2 million people (∼ 8% of the world population) (WHO) (WHO, 2023) are not treated with technological tools and quantifiable multisensory rehabilitation training. Thus, the multisensory information afforded by *iReach* represents a major leap toward i) the development of a reliable and quantitative system to quantify infants’ ability to react to unimodal and bimodal audio and tactile events and to evaluate and monitor their spatial and bodily impairments, ii) the rehabilitation of infants’ impaired mechanisms through non-invasive multisensory technology, and iii) an early intervention considering the specific sensitive period. Through its early and scientific-based appropriate intervention, *iReach* will enhance the wellbeing of visually impaired patients and their families to improve their quality of life and social inclusion (Cappagli et al., 2017). The use of *iReach* might be extended also to visually impaired children, adults, and the elderly that, like infants, might need a very natural and simple language, boosting their neural plasticity. It might have also the potential for rehabilitative purposes in individuals with sensory implants (e.g., retinal and acoustic implants) as well as unilateral neglect, cognitive impairment, and motor deficits.
(3) Impact on neuroscience. *iReach* will pave the way to increase our knowledge of multisensory integration at different cortical levels, space and body representation mechanisms, and motor skills. This synergy would be a breakthrough in neuroscience since the system will allow direct exploration of the role of vision in the multisensory processing development from infancy and to define the mechanisms behind it. Recording EEG and behavioral data simultaneously will, for the first time, allow to link the sensory modulation of space and body representation, underlying cortical circuits, and visual impairments. *iReach* will be widely used in research laboratories around the world to address basic science questions about multisensory processing. Notably, these basic science results are a prerequisite to determine the sensitive periods for developing spatial and bodily representations and for functional impairment/recovery in visually impaired infants.

## Conclusion

A wide variety of products for blind people have already been developed and distributed (for further information).^2^ Many of these are low-level technology products, like those used by sighted individuals but having been adapted for blind users. Other products provide essential information to blind individuals (e.g., smart canes for navigation and Braille displays); notably, such products are all essentially different from *iReach*. At the research level, a great deal of effort has been put into developing sensory substitution devices (SSDs) that aim to convey visual information through different modalities. However, in general, the wide amount of tactile or acoustic signals coming from these devices tends to overload the capacity of blind individuals to process this information. As a result, sensory substitution devices have not been widely adopted by visually impaired people. Unlike SSDs, the *iReach* device is a rehabilitation-oriented device. The goal of *iReach* is to increase the autonomy of the individual, not to convey visual information through a different sensory modality. The aim is not to force our brains to adapt to the language of a device, which sometimes provides a redundant and heavy amount of information just because it is possible to get it with a plethora of sensors; rather, the aim of the *iReach* is to use a natural, ecological and simple language to boost a sophisticated device that will stimulate the brain to recover a more efficient representation of the surrounding space and of the body.

## Data Availability

The original contributions presented in this study are included in this article/supplementary material, further inquiries can be directed to the corresponding author.

## Footnotes

1
https://www.thelancet.com/

2
https://www.afb.org/prodmain.asp
